# Detecting the most critical clinical variables of COVID-19 breakthrough infection in vaccinated persons using machine learning

**DOI:** 10.1177/20552076231207593

**Published:** 2023-11-05

**Authors:** Olawande Daramola, Tatenda Duncan Kavu, Maritha J Kotze, Oiva Kamati, Zaakiyah Emjedi, Boniface Kabaso, Thomas Moser, Karl Stroetmann, Isaac Fwemba, Fisayo Daramola, Martha Nyirenda, Susan J van Rensburg, Peter S Nyasulu, Jeanine L Marnewick

**Affiliations:** 1 Department of Information Technology, Faculty of Informatics and Design, 70683 Cape Peninsula University of Technology, Cape Town, South Africa; 2 Division of Chemical Pathology, Department of Pathology, Faculty of Medicine and Health Sciences, Stellenbosch University, Cape Town, South Africa; 3 Division of Chemical Pathology, Department of Pathology, National Health Laboratory Service, Tygerberg Hospital, Cape Town, South Africa; 4 Applied Microbial and Health Biotechnology Institute (AMHBI), 146301 Cape Peninsula University of Technology, Cape Town, South Africa; 5 Department of Biomedical Sciences, Faculty of Health and Wellness Sciences, 210360 Cape Peninsula University of Technology, Cape Town, South Africa; 6 521340 St. Pölten University of Applied Sciences, St. Pölten, Austria; 7 School of Health Information Science, 175083 University of Victoria, Victoria, BC, Canada; 8 Division of Epidemiology and Biostatistics, 121470 Faculty of Medicine and Health Sciences, Stellenbosch University, Cape Town, South Africa

**Keywords:** Machine learning, vaccination, COVID-19, breakthrough infection, Pfizer vaccine, J&J vaccine, Explainable AI, XGBoost, deep multilayer perceptron, logistic regression

## Abstract

**Background:**

COVID-19 vaccines offer different levels of immune protection but do not provide 100% protection. Vaccinated persons with pre-existing comorbidities may be at an increased risk of SARS-CoV-2 breakthrough infection or reinfection. The aim of this study is to identify the critical variables associated with a higher probability of SARS-CoV-2 breakthrough infection using machine learning.

**Methods:**

A dataset comprising symptoms and feedback from 257 persons, of whom 203 were vaccinated and 54 unvaccinated, was used for the investigation. Three machine learning algorithms – Deep Multilayer Perceptron (Deep MLP), XGBoost, and Logistic Regression – were trained with the original (imbalanced) dataset and the balanced dataset created by using the Random Oversampling Technique (ROT), and the Synthetic Minority Oversampling Technique (SMOTE). We compared the performance of the classification algorithms when the features highly correlated with breakthrough infection were used and when all features in the dataset were used.

**Result:**

The results show that when highly correlated features were considered as predictors, with Random Oversampling to address data imbalance, the XGBoost classifier has the best performance (F1 = 0.96; accuracy = 0.96; AUC = 0.98; G-Mean = 0.98; MCC = 0.88). The Deep MLP had the second best performance (F1 = 0.94; accuracy = 0.94; AUC = 0.92; G-Mean = 0.70; MCC = 0.42), while Logistic Regression had less accurate performance (F1 = 0.89; accuracy = 0.88; AUC = 0.89; G-Mean = 0.89; MCC = 0.68). We also used Shapley Additive Explanations (SHAP) to investigate the interpretability of the models. We found that *body temperature, total cholesterol, glucose level, blood pressure, waist circumference, body weight, body mass index (BMI), haemoglobin level, and physical activity per week* are the most critical variables indicating a higher risk of breakthrough infection.

**Conclusion:**

These results, evident from our unique data source derived from apparently healthy volunteers with cardiovascular risk factors, follow the expected pattern of positive or negative correlations previously reported in the literature. This information strengthens the body of knowledge currently applied in public health guidelines and may also be used by medical practitioners in the future to reduce the risk of SARS-CoV-2 breakthrough infection.

## Introduction

The effect of the COVID-19 pandemic on healthcare systems all over the world has been devastating.^1^ As a result, various clinical intervention methods have been employed in detecting, diagnosing, and prognosticating of COVID-19 cases, including the development and worldwide administration of vaccines.^2^ However, vaccines do not provide 100% immunity, meaning some people still test COVID-19 positive even after vaccination. A breakthrough infection is present when someone who completed vaccination still gets infected.^3^ Lately, the use of Artificial Intelligence (AI) methods for tackling the challenges around diagnosing and treating COVID-19 has received significant attention. Some of the efforts so far reported include the application of machine learning (ML) to predict or detect SARS-CoV-2 breakthrough infections.^2–7^ South Africa recorded the highest number of COVID-19 cases in Africa. According to Daramola et al.,^8^ AI-enabled decision-making for treating COVID-19 can enhance healthcare quality. However, this is not common, particularly in many African countries.

The present paper demonstrates the application of ML algorithms to identify the most critical variables for predicting COVID-19 breakthrough infection. We used a dataset that contains symptoms and feedback from apparently healthy volunteers with cardiovascular risk factors for this investigation. We selected three supervised learning classifiers for our predictive analytics: Deep Multilayer Perceptron (Deep MLP), Extreme Gradient Boosted Trees (XGBoost), and Logistic Regression (LR). This is because Deep MLP and XGBoost rank among the best ML algorithms when dealing with tabular datasets based on evidence from the literature and ML competitions like Kaggle,^9,10^ while on many occasions in healthcare research, the LR is used as a baseline to compare with more complex ML algorithms.^11^

The multilayer perceptron (MLP) is an Artificial Neural Network (ANN) model that can be used to solve classification and regression tasks. A Deep MLP is a feedforward ANN with multiple (more than one) hidden layers fully connected in a dense architecture. The XGBoost is an ensemble learning model that uses gradient boosting to solve classification and prediction tasks. In ML, boosting entails combining a set of weak learners and aggregating their predictions to obtain improved prediction accuracy. The XGBoost algorithm grows a set of classification and regression trees (CART) sequentially to improve classification performance during subsequent iterations. LR is a statistical learning algorithm that can determine if a dependent categorical outcome belongs to a particular class based on a set of independent variables. LR entails first computing a linear regression value from the data and then applying a logistic function (Sigmoid function) (0, 1)) to determine the probability that the linear regression value belongs to a specific class (0 or 1).

This paper has three objectives. The first is to demonstrate how state-of-the-art ML algorithms can be applied to predict SARS-CoV-2 breakthrough infection. The second is to investigate how the application of two data balancing techniques, Random Oversampling Technique (ROT) and the Synthetic Minority Oversampling Technique (SMOTE), on a highly imbalanced dataset affects the performance of ML models. The third is to identify the variables with the highest prognostic value regarding COVID-19 breakthrough infection. Data bias is one of the concerns of data scientists when training ML models and can come from class imbalance. Class imbalance is present in many real-world classification datasets. It connotes a disproportionate distribution of the number of examples of the different classes in the problem.^12^ With respect to the dependent variable, if there is an over-representation of certain values, the model will tend to predict those values well at the expense of the underrepresented values. Therefore, in this study, statistical methods like ROT and SMOTE were used to solve the problem of data imbalance.^13^ The data used in this study had proportionately large instances of ‘no breakthrough infection’ (161 out of 203), so if the data imbalance is not addressed using ROT or SMOTE, the models’ predictions would be more biased towards no breakthrough infection as an outcome than breakthrough infection. Thus, in this study, we performed experiments to compare the performance of the three classification algorithms, Deep MLP, XGBoost, and LR, to predict breakthrough infection when (1) an imbalanced dataset and instances of highly correlated features and all features in the dataset are used; and (2) when ROT and SMOTE were applied to obtain a balanced dataset, for instances of highly correlated features, and all features in the dataset.

So far, few cases of forecasting breakthrough infections, especially using ML, have been reported in the literature. Therefore, we briefly highlight some related work. A time-series ML model was designed by Rasheed and colleagues,^14^ which predicted the time series of new daily positive cases, severe cases, hospitalised cases, and deaths. The authors acquired data from regions with high rates of vaccination to examine the effect of vaccination. The study also considered the effectiveness of vaccination, vaccination protection waning effect ratio, and infectivity of different variants. The study provided an effective approach to forecasting COVID-19 cases based on the waning effect of the vaccination per population. The authors found that the waning effect differed per each country of their case study. However, the authors did not identify the main factors that could increase the probability of breakthrough infection in patients.

Wang and colleagues^6^ looked into emerging vaccine-breakthrough SARS-CoV-2 (viz. COVID-19) variants. They used work that involved using deep learning (DL) to reveal the SARS-CoV-2 evolution mechanism and forecast emerging vaccine-breakthrough variants. The result showed that infectivity-strengthening variants were the main mechanism for viral evolution, while vaccine-escape variants became a dominating viral evolutionary mechanism among highly vaccinated populations. The authors also demonstrated that the Lambda variant is as infectious as the Delta but is more vaccine-resistant. Wang et al.^15^ proposed a ML framework to predict COVID-19 infection/reinfection severity levels from salivaomics data. The ANN model achieved an accuracy of 0.85, adjudged to be computationally efficient and useful in a clinical setting because results can be generated within a few milliseconds of CPU time. Wedlund and Kvedar^16^ presented an editorial on a widely applicable tool that allows clinicians to predict uninfected individuals who might benefit most from COVID-19 vaccination. The authors observed that the model could help allocate therapies and equipment to those most at-risk, maximising survival.

Liao et al.^17^ proposed a predictive model incorporating mutational information to predict COVID-19 variants of concern (VOC). The authors created a DL prediction framework based on VOC, which includes VOC-Long Short-Term Memory (LSTM), VOC-Gated Recurrent Units (GRU), and VOC-Bidirectional Long Short-Term Memory (BILSTM) algorithms. The study used a time series dataset containing daily newly confirmed cases in Italy, South Korea, Russia, Japan, and India from 14 April 2021 to 3 July 2021 and VOC variant information. The result showed that VOC-LSTM exhibited superior performance compared to other algorithms in its prediction accuracy. Kumar et al.^18^ proposed a novel RNN Convolutional Residual Network (RNNCON-Res) to predict the spread of Coronavirus variants. The proposed model leverages the power of Res-RNN with some modifications, Gated Recurrent Unit (GRU) and LSTM units, to handle the long-term dependencies. The RNNCON-Res recorded an accuracy of 91% in country-level prediction 20 days ahead, which was better than the state-of-the-art methods. Ahamad and colleagues^19^ investigated the causes of COVID-19 postvaccination adverse events (death, reinfection, and hospitalisation) in patients using predictive modelling. Six algorithms were used, which are decision tree (DT) and random forest (RF), support vector machine (SVM), gradient boosting machine (GBM), extreme gradient boosting machine (XGB), and light gradient boosting machine (LGBM). A dataset of patients’ adverse reactions after vaccination was used for the study. The result showed that RF had an accuracy of 100%, while the other algorithms had an accuracy score above 90%. Also, patients with the highest risk for adverse postvaccination reactions include older patients (60 years and above), gender, chronic obstructive pulmonary disease (COPD), hypertension, those having allergic conditions, those taking other medications (particularly immunosuppressive medications), and those with comorbidities such as history of type-2 diabetes, hypertension, or heart disease disorders. In addition, postvaccination symptoms such as hospital stay duration, pyrexia, headache, dyspnoea, chills, fatigue, different kinds of pain and dizziness, rash, and physical disability were found to be closely associated with adverse reactions.

Also, Ebrahimi et al.^20^ developed a ML model for predicting overall survival (OS) among reinfected COVID-19 patients. Two ML algorithms were applied – elastic-net regularised Cox-adjusted PH model and backward stepwise elimination – to a dataset of 283 reinfected COVID-19 patients admitted to 26 medical centres in Iran. The result shows that the in-hospital mortality rate among the reinfected COVID-19 patients was 9.5%, while the mortality rate among the intubated patients was 83.5%. Using the Kaplan-Meier approach, the OS at 95% confidence interval (CI) that was obtained for days 7, 14, and 21 were 87.5%, 78.3%, and 52.2%, respectively. The findings also show that factors such as Emergency Medical Services (EMS) transfer, profound hypoxemia, increased serum creatinine, and increased white blood cell (WBC) count reduced the OS of reinfected COVID-19 patients. Afrash et al.^21^ investigated the most critical features of COVID-19 readmission due to reinfection and compared the prediction performance of six ML algorithms. These are XGBoost, Hist Gradient Boosting (HGB), Bagging classifier, Multi-Layered Perceptron (MLP), SVM (kernel: linear), and SVM (kernel: RBF). A dataset of 870 re-admitted COVID-19 patients was used. Using the LASSO feature selection algorithm, 14 out of 42 features were selected as the most relevant predictors. The result showed that XGBoost had the best performance out of the seven algorithms, with an average accuracy of 91.7%, F1-score of 91.8%, and AUC of 91%. The study also found that COVID-19 status, ICU admission, and oxygen therapy were the features most associated with readmission of COVID-19 patients, while age and solid metastatic tumour were the least associated. Chen and colleagues^22^ claimed an accurate model to predict COVID-19 breakthrough infection is still lacking. Hence, they constructed a visualised nomogram using the stepwise multivariate LR algorithm. The study used a dataset of 6189 vaccinated individuals, consisting of COVID-19 test-positive cases (*n* = 219) and test-negative controls (*n* = 5970) during the outbreak of the Delta variant in September 2021 in Xiamen and Putian cities, Fujian province of China. At a 95% CI, the result showed that the nomogram had an area under the curve (AUC) score of 81.9% for the training dataset and 83.8% for the validation set. The decision curves generated by the nomogram to determine the probability of COVID-19 breakthrough infection had an optimal agreement with actual clinical observation.

Our literature review revealed that few cases of application of ML for predicting COVID-19 breakthrough infection in patients have been reported so far. More so, studies focusing on predictive modelling of COVID-19 breakthrough infection cases from the African context are rare in the literature. Given this context, our study makes the following contributions:

- It demonstrates how ML algorithms can predict COVID-19 breakthrough infection based on a dataset containing symptoms and feedback from apparently healthy volunteers with cardiovascular risk factors.
- It demonstrates how an explainability model like SHAP can be applied to identify critical variables with prognostic value for predicting COVID-19 breakthrough infection.
- It provides a comparative analysis of the performance and interpretability of Deep MLP, XGBoost, and LR when ROT and SMOTE are used for data balancing in predicting COVID-19 breakthrough infection.
- It offers the first report on the application of ML for predicting COVID-19 breakthrough infection from the African context, which makes it an empirical contribution to the extant literature.

The rest of this paper is organised as follows: The Methods section presents the methodology adopted for the study, and the Results section presents the results. In the Discussion section, we discuss the results, while the paper concludes in the Conclusion section with a summary and plan for future work.

## Methods

Ethical approval for the study was obtained from the Faculty of Health and Wellness Sciences Research Ethics Committee (HWREC) of the Cape Peninsula University of Technology (CPUT) for the Rooibos, Heart and Cognitive Health study (CPUT/HW-REC 2017/H9-extension). Also, ethical approval for the data analytics experimentation on health-related data was obtained from the Research Ethics Committee of the Faculty of Informatics and Design of the CPUT (30/Daramola/2021). A data-sharing agreement was in place between authors OD (corresponding author) and JLM (Lead, Rooibos, Heart and Cognitive Health study) on 24 June 2022.

### Description of the dataset

The data was collected from an intervention study conducted within the City of Cape Town region in South Africa. The participants in the study were not selected by COVID-19 status but were included in the study based on the presence of cardiovascular disease (CVD) risk factors. Written informed consent was obtained from each study participant before the study commenced, following an information session attended by all the participants. After explaining the objectives of the study, an opportunity was provided for asking questions about the study before providing written informed consent.

Clinical sample collection started on 8 November 2021 and ended on 8 April 2022. The prevalent COVID-19 variants in South Africa (Western Cape area) during the time of the study was the Omicron variant with BA.1, BA.2, and BA.3, but with BA.1 responsible for the most infections during South Africa's fourth epidemic wave.^23^ BA.2 became dominant from the middle of January 2022, with BA.4 and BA.5 originating since the middle of December 2021. Davies et al.^24^ claimed that disease severity caused by BA.4 and BA.5 was similar to BA.1 taken within the context of developing immunity against SARS-CoV-2 caused by prior infection and vaccination, both of which were highly protective. Although the vaccine type was recorded for each participant, our focus in this particular study was on the vaccination status of the participants, not on the particular type of vaccine they obtained. The demographic and clinical characteristics of the study participants are shown in Table 1.

**Table 1.** Demographic and clinical characteristics of the study participant profile.

Attribute	Data value
Gender	Females = 192 Males = 65
Age (years)	46 ± 11
BMI (body mass index)	30.49 ± 7.37
Total cholesterol (mmol/L)	5.38 ± 0.96
Systolic Blood Pressure (BP) (mmHg)	134 ± 19
Diastolic Blood Pressure (BP) (mmHg)	85 ± 12
Glucose (mmol/L)	4.55 ± 1.78
Smokers (%)	30%
Primary vaccine received (Yes/No)	Yes = 203 No = 54
Booster vaccine received (Yes/No)	Yes = 52 No = 205
Breakthrough SARS-COV-2 infections (Yes/No)	Yes = 42 No = 161

The dataset consists of 60 features and 257 rows (records). There is one target variable – breakthrough infection or not. A snapshot of the dataset is presented in Tables 2 and 3. The dataset was imbalanced regarding the target outcome (breakthrough infection or not) in the ratio of 42:215.

**Table 2.** Description of categorical features in the dataset.

S/n	Attribute	Feature name	Data value	Frequencies
1	Gender	Gender	Females / Males	192, 65
2	Marital_status	Marital status	Never Married / Living together / Married / Divorced / Widow	75, 13, 130, 31, 8
3	Primary vaccine received	I have been vaccinated with either Pfizer (2 doses) or J&J (1 dose)	Yes / No	203, 54
4	Booster vaccine received	If booster vaccine was received	Yes / No	52, 205
5	HLE	Highest level of Education	Primary school/Metric-grade 12 / Tertiary education / Postgraduate education	18, 170, 55, 14
6	Smoking	Smoking	No /Yes / Previous	141, 78, 38
7	Physical_Activity	Physical Activity	Yes / No	189, 68
8	HPT	Hypertension?	Yes / No / Unsure	229, 10, 18
9	Low/high_Blood_sugar	Blood sugar levels	Yes / No / Unsure	230, 5, 22
10	Fam_hx_CVD	Family history of cardiovascular disease	Yes/No/Unsure	106, 138, 13
11	Kidney_problems	Kidney problems?	No / Yes	255, 2
12	Liver_problems	Problem with the Liver?	No / Yes	256, 1
13	Blood_pressure	Blood pressure	High / Normal / Mild_Hypertension / Optimal / Moderate_Hypertension / Severe_Hypertension	117, 56, 51, 18, 13, 2
14	First_Language	First language	Afrikaans /English /Xhosa /Others	121, 72, 38, 26
15	Weight_Category	Weight category	Obese/ Overweight / Normal /Under Weight	128, 70, 56, 3
16	Occupation	Occupation	Employed / Unemployed	193, 64
17	Hazardous_exposure	Have been exposed to hazardous substances	No/ Yes	224, 33
18	Alcohol	Drink Alcohol?	No / Yes / Occasionally	162, 94, 1
19	Symp_Cold _b	Feeling cold at baseline?	No / Yes/ Hot	255, 1, 1
20	Symp_Cough _b	Coughing at baseline?	No / Yes	254, 3
21	Symp_Headache_b	Feeling headache at baseline	No / Yes	253, 4
22	breathlessness_b	Failing to breathe at baseline _	No / Yes	256, 1
23	Taste_Smell_b	Can’t taste at baseline?	No / Yes	255, 2
24	Close_to_Someonewith_Covid _b	I have been close with someone with COVID-19 at baseline.	No/Yes	256, 1
25	Positive_Before _b	Have you tested positive before at baseline?	No / Yes	223, 34
26	COVID-19_comorbidities_b	Are you under treatment for any chronic disease condition (the name of the disease is not required)?	No / Yes	256, 1
27	Vaccinated_SARS-CoV-2_Baseline	Have been vaccinated against COVID	Yes / No	219, 38
28	COVID-19_Screening_Results_b	Test results at baseline	Positive / Negative / NS = NR / Inconclusive	141, 80, 30, 6
29	Symp_Cold_i	Feeling cold at intervention?	No / Yes / Warm	253, 3, 1
30	Symp_Cough_i	Coughing at intervention?	No / Yes	254, 3
31	Symp_Headache_i	Feeling headache at intervention	No / Yes	252, 5
32	breathlessness_i	(Failing to breathe at intervention?	No / Yes	254, 3
33	taste_Smell_i	Can’t taste at intervention?	No / Yes	257
34	Close_to_Someonewith_Covid_i	I have been in close contact with someone with COVID at intervention.	No / Yes	257
35	Positive_Before_i	Have you tested positive before intervention?	No / Yes	207, 50
36	COVID-19_comorbidities_i	Have some comorbidities at intervention?	No / Yes	251, 6
37	Vaccinated_SARS-CoV-2_i	Have been vaccinated against COVID at intervention?	Yes / No	212, 45
38	COVID-19_Screening_Results_i	Test results at baseline at intervention?	Negative / Positive / NS = NR / inconclusive	144, 83, 56, 3
39	Breakthrough SARS-COV-2 infections (Outcome variable)	Had breakthrough infection	Yes / No	42, 161

**Table 3.** Description of numeric features in the dataset.

S/n	Variable	Field name	Range	Mean (STD)	Standard deviation
40	Weight	Weight	43–178	80.96	19.23
41	Height	Height	1–2	1.98	0.12
42	Waist_circumference	Waist circumference	62–149	98.29	15.06
43	BMI_x	Body Mass Index at Baseline	16–59	30.49	7.37
44	HB	Haemoglobin	7–21	14.00	2.41
45	Age	Age	30–77	46.18	10.62
46	PA/week	Physical activity per week	1–3	0.88	0.71
47	PA_level	Physical Activity Level	1–2	1.23	0.42
48	High_Chol	High Cholesterol	1–3	1.38	0.69
49	BMI_y	Body Mass Index at Intervention	16–59	30.52	7.36
50	Glucose_Level(mmol/L)	Blood Glucose Level	1.7–16.9	4.55	1.78
51	Chol	Total Cholesterol level	3.89–8.2	5.38	0.96
52	Temp_b	Temperature at baseline	35.6–36.9	36.33	0.28
53	Temp_i	Temperature at Intervention	35.1–37.8	36.31	0.38

### Data preprocessing

**Figure 1.**
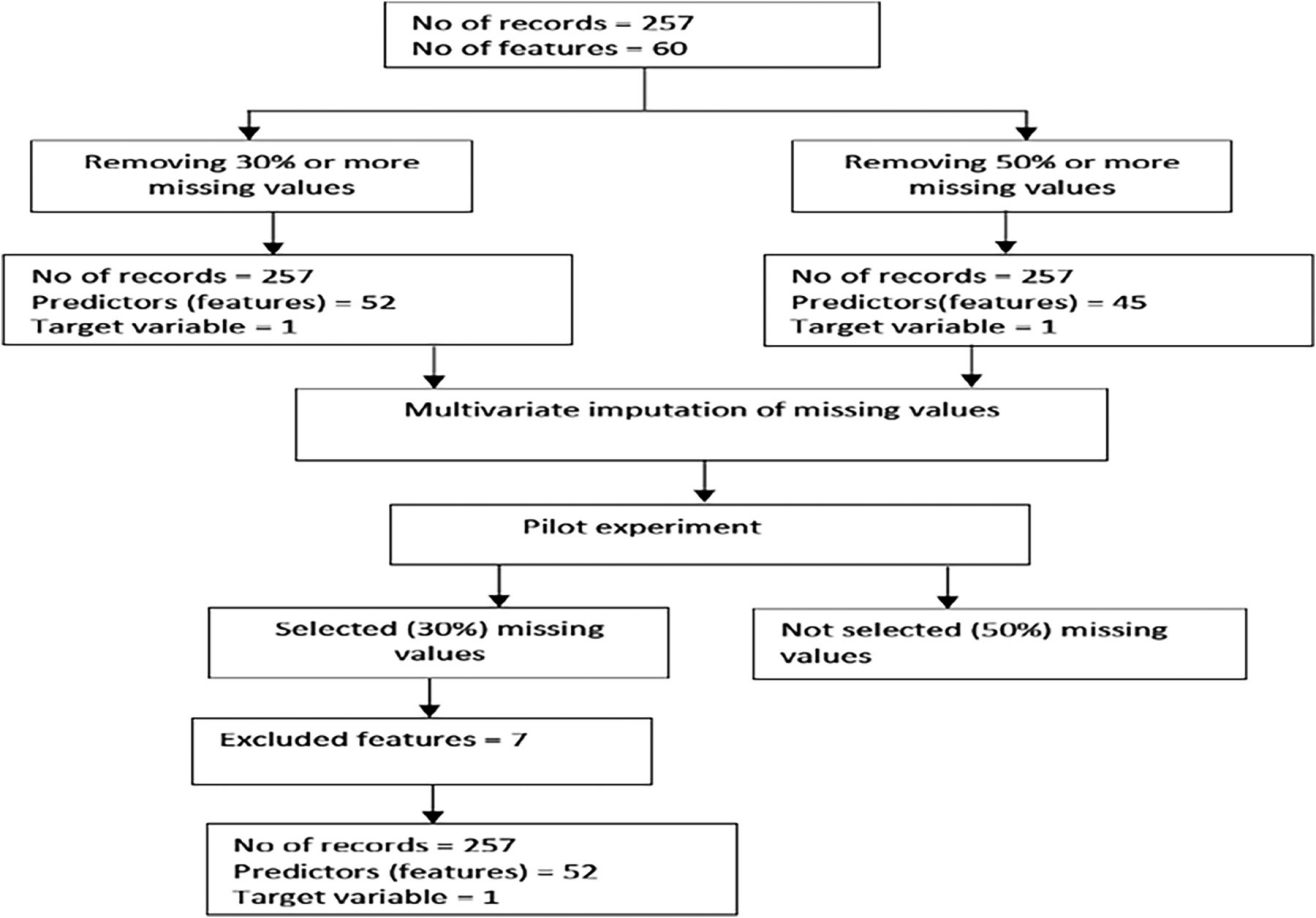
Selection of records for experimentation.

The dataset contained some missing values; therefore, the multivariate imputation technique based on an underlying XGBoost regressor algorithm was applied to fix missing values. To do this, we experimented with the case of removing features (variables) with 30% vs. 50% missing values. Working with 30% missing values produced better overall results (see Appendix 1); hence, we removed features with 30% or more missing values and those considered redundant due to duplication. After preprocessing, we used 53 variables (52 independent variables and one dependent variable) and 257 rows for our experimentation. The exclusion criteria applied to obtain the variables and records used for our experimentation are shown in Figure 1.

### Experiments

**Figure 2.**
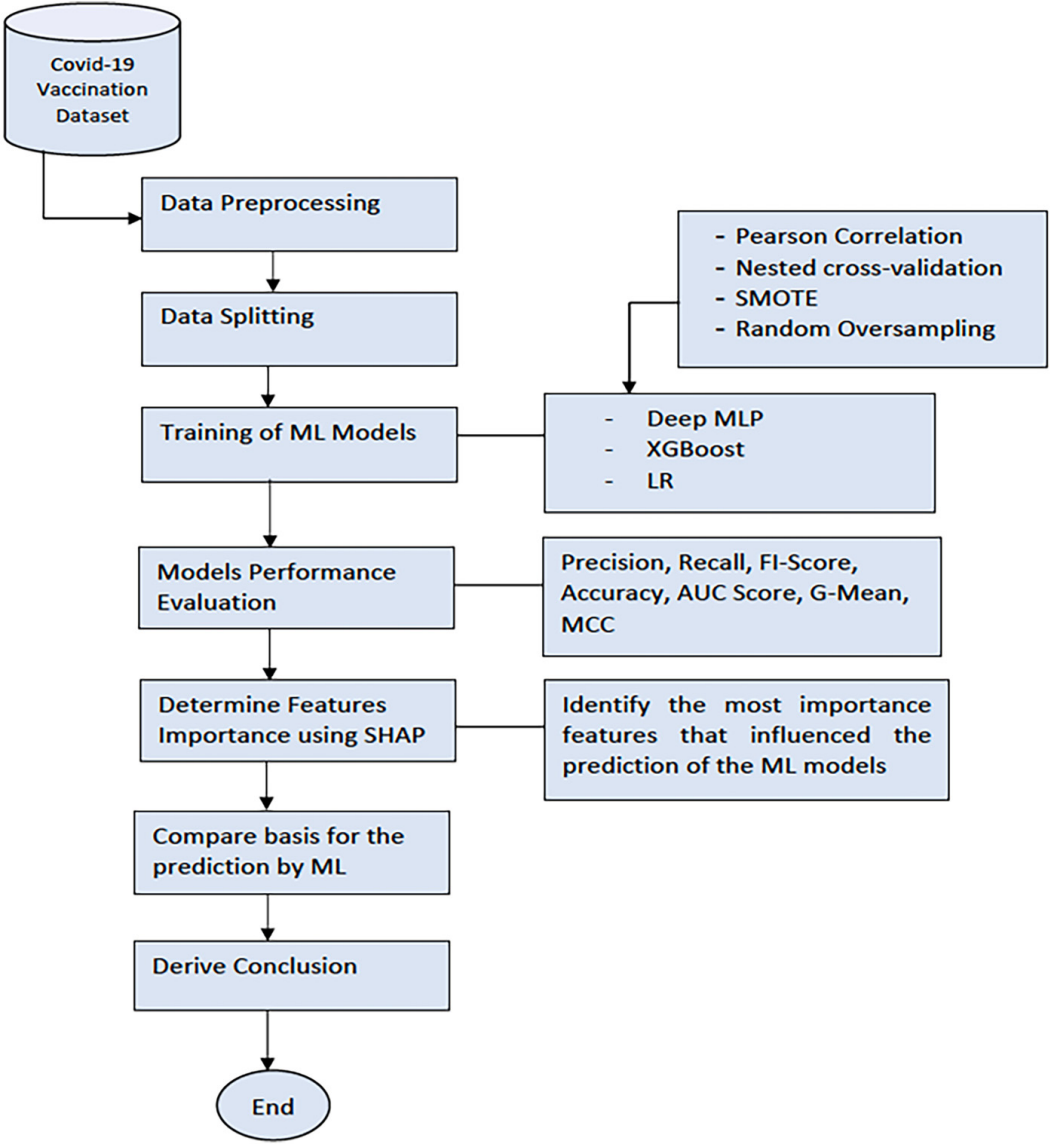
The workflow of the experimentation process.

Figure 2 shows the process workflow of our experimentation. Firstly, data were collected from the recruitment site at the CPUT, Cape Town, South Africa. Next, we applied data preprocessing techniques to clean up the data and transform the data to numerical form (the numeric data points were scaled (0–1) using the standard scaler normalisation function. The categorical variables were encoded using the leave one-hot encoding. We then split the dataset into the training and testing sets. The training set was used to train the Deep MLP, XGBoost, and LR model. Later, we evaluated the performance of the ML models on the test set using standard classification metrics. Finally, we benchmarked the performance of the models using the F1-score, accuracy, AUC score, Geometric mean (G-Mean), and the Matthews correlation coefficient (MCC). The Classification Report method of the Scikit Learn framework^3^ was used to generate the F1-score, recall, accuracy, and precision of the ML models. The report also contains the macro average (the average score across all classes for precision, recall, and F1) and the weighted average (the mean value of a metric per class (e.g. F1, recall, precision) while considering the support of each class).

After this, we applied the SHAP model, a unified framework for interpreting predictions by ML models, to investigate the model interpretability.^25^ SHAP can determine the level of the global importance of each feature to the prediction generated by a ML model. SHAP is a mathematical method based on game theory that can explain any ML model's predictions by calculating each feature's contribution to the prediction.^26^ We then derived our conclusion by selecting the most important features based on the SHAP values generated by the ML model that had the best performance (XGBoost).

### Models training

The three ML models, Deep MLP, XGBoost, and LR, were trained using optimal parameters generated by the nested cross-validation and Grid search function in Scikit Learn.^3^ The optimal parameters automatically selected for training the models are shown in Table 4. After determining the best option that produced the best performance, we applied SHAP to the selected ML models to determine the most important features that influence breakthrough infection.

**Table 4.** Hyperparameters for training the ML models.

XGBoost: Best parameters	MLP: Best parameters	LR: Best parameters
n_estimators = 300, max_depth = 4, learning_rate = 0.01, min_child_weight = 1, gamma = 0, subsample = 0.8, colsample_bytree = 0.8, objective = ‘binary:logistic’, nthread = 4, scale_pos_weight = 1, seed = 27	Input layer units = 49 Hidden layer 1: units = 69 Hidden layer 2: units = 73 Output layer units = 1 Activation: Input layer (relu) Hidden layer 1 (relu) Hidden layer 2 (relu) Outer layer (sigmoid) loss = binary_crossentropy, optimizer = RMSprop batch_size = 10 epochs = 100 outer_cv = StratifiedKFold (n_splits = 5, shuffle = True, random_state = 42) inner_cv = StratifiedKFold (n_splits = 3, shuffle = True, random_state = 42) dropout_rate: 0.5 weight_constraint: 5.0	C: 10.0, penalty: l2

## Results

The dataset used for our study contains anonymised data from 257 participants, of which 203 (78.9%) were vaccinated. The dataset consists of several aspects, such as:

- Socio-demographic and lifestyle characteristics (e.g. age, gender, occupation, smoking status, alcohol use, drug use)
- Exposure history and symptoms (previous contact with infected persons or places; symptom types, severity score, etc.)
- Vaccination status (Yes/No)
- Blood components (glucose level, total cholesterol level)
- Body status (body mass index (BMI), blood pressure, waist circumference, weight, temperature)
- COVID-19 test results at (baseline and after a 12-week dietary antioxidant intervention)

We considered six options in our experimentation:

- Highly correlated features and an imbalanced dataset
- All features in the dataset and imbalanced dataset
- Highly correlated features + SMOTE
- All features in the dataset + SMOTE
- Highly correlated features + ROT
- All features in the dataset + ROT

After determining the best option that produced the best performance, we applied SHAP^26^ to assess the interpretability of selected models. Our objective was to determine the most significant features that influence the prediction of breakthrough infection.

We evaluated the performance of ML models when the six options were implemented using five standard metrics that are deemed relevant. The selected metrics are defined as follows:

- F1-score: The harmonic mean of precision and recall gives a more balanced description of model performance. It is a value between 0 and 1. The F1-score is a suitable metric for assessing model performance for an imbalanced dataset. F1Score=(2×PR/P)+R . F1-score is interpreted as follows: F1 > 0.9 (very good); 0.8–0.9 (good); 0.5–0.8 (okay); <0.5 (not good).
- AUC score: The AUC measures how well a classifier can distinguish between classes and is used as a summary of the receiver operating curve (ROC). The AUC score is rated as follows: excellent (0.9–1), good (0.8–0.9), fair (0.7–0.8), poor (0.6–0.7), and failed (0.5–0.6).
- G-Mean: The G-Mean considers the relative balance of the classifier's performance on both the minority and majority classes.^26^ It is defined as a function of the classifier's sensitivity and specificity. The value of G-Mean is in the range of 0–1, where a value that is closer to 1 is indicative of superior performance. The G-Mean score is rated as follows: excellent (0.9–1), good (0.8–0.9), fair (0.7–0.8), poor (0.6–0.7), and failed (0.5–0.6).
- MCC: The MCC is a measure from the field of Bioinformatics, where class imbalance occurs very often. It is an adaptation of the Pearson correlation coefficient to evaluate the correlation in confusion matrices. MCC ranges from −1 (when the classification is always wrong) to 0 (when it is no better than random) to 1 (when it is always correct).^27^
- Accuracy: the percentage of prediction that is correct. It is measured by dividing the number of correct predictions by the total number of predictions. Accuracy is not a good metric to assess a ML model when the dataset is imbalanced.^28^

### Highly correlated features using an imbalanced dataset

All the models were trained using grid search to identify the optimal hyperparameters for training and nested cross-validation.

**Figure 3.**
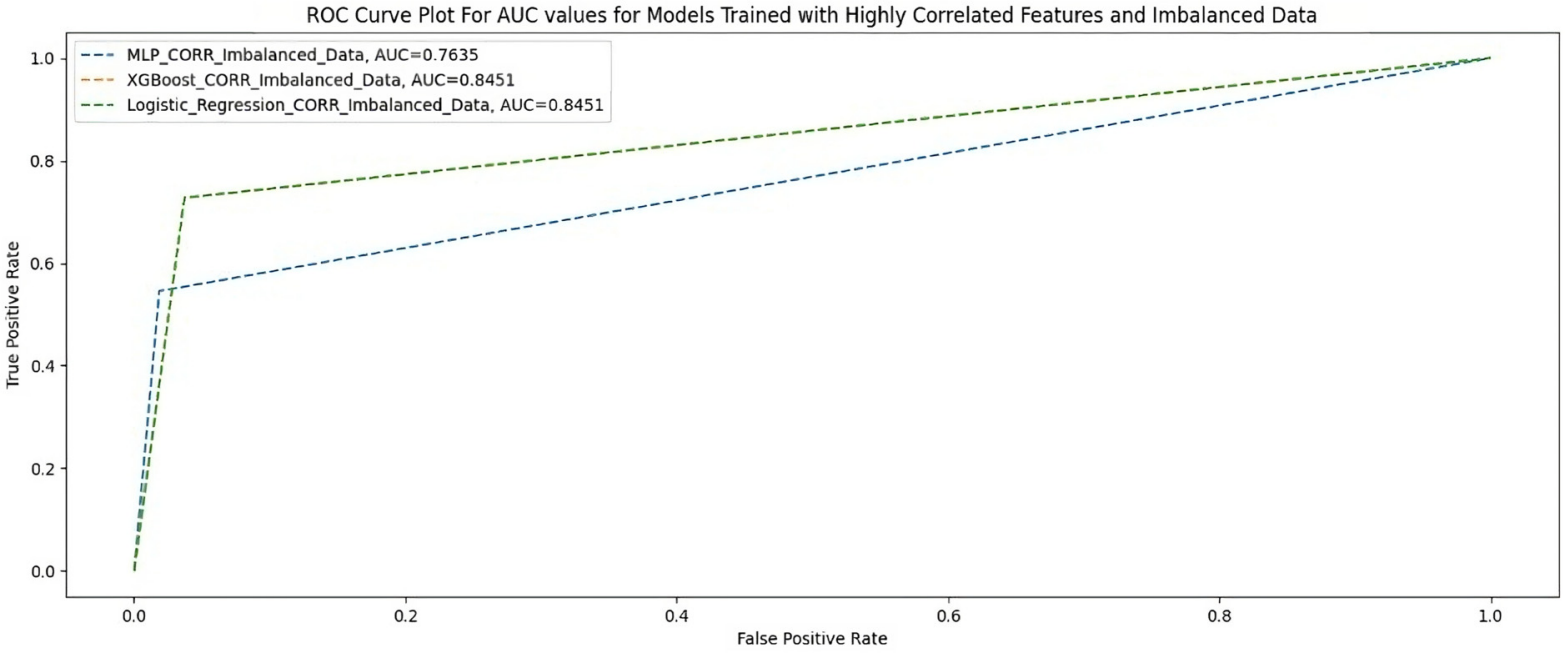
AUC scores for models trained with highly correlated features and imbalanced dataset.

Table 5 and Figure 3 show the performance of the three ML models measured using F1-score, AUC-score, G-Mean, MCC, and accuracy when trained with 23 highly correlated features with the breakthrough infection using an imbalanced dataset.

**Table 5.** Highly correlated features and an imbalanced dataset.

Measures	Deep MLP	XGBoost	Logistic regression
F1-Score	0.84	0.92	0.92
G-Mean	0.59	0.84	0.84
MCC	0.42	0.72	0.72
AUC-Score	0.66	0.85	0.85
Accuracy	0.86	0.92	0.92

### Highly correlated features + random oversampling

**Figure 4.**
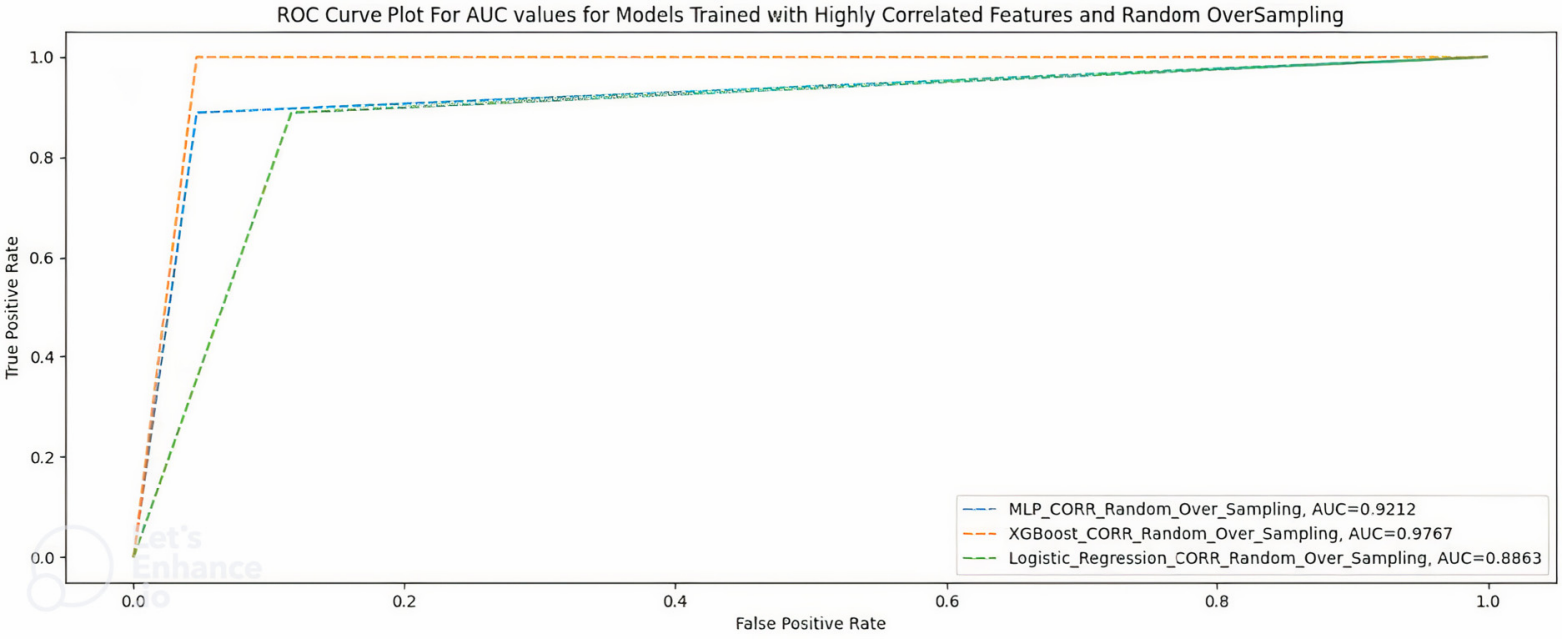
AUC scores for models trained with highly correlated features + random oversampling.

Table 6 shows the performance of the three ML models when trained with 23 highly correlated features with breakthrough infection with ROT. Figure 4 shows the AUC scores of the three models when trained under the same condition.

**Table 6.** Highly correlated features + random oversampling.

Measures	Deep MLP	XGBoost	Logistic regression
F1-score	0.94	0.96	0.89
G-Mean	0.70	0.98	0.89
MCC	0.42	0.88	0.68
AUC score	0.92	0.98	0.89
Accuracy	0.94	0.96	0.88

### Highly correlated features +  SMOTE

**Figure 5.**
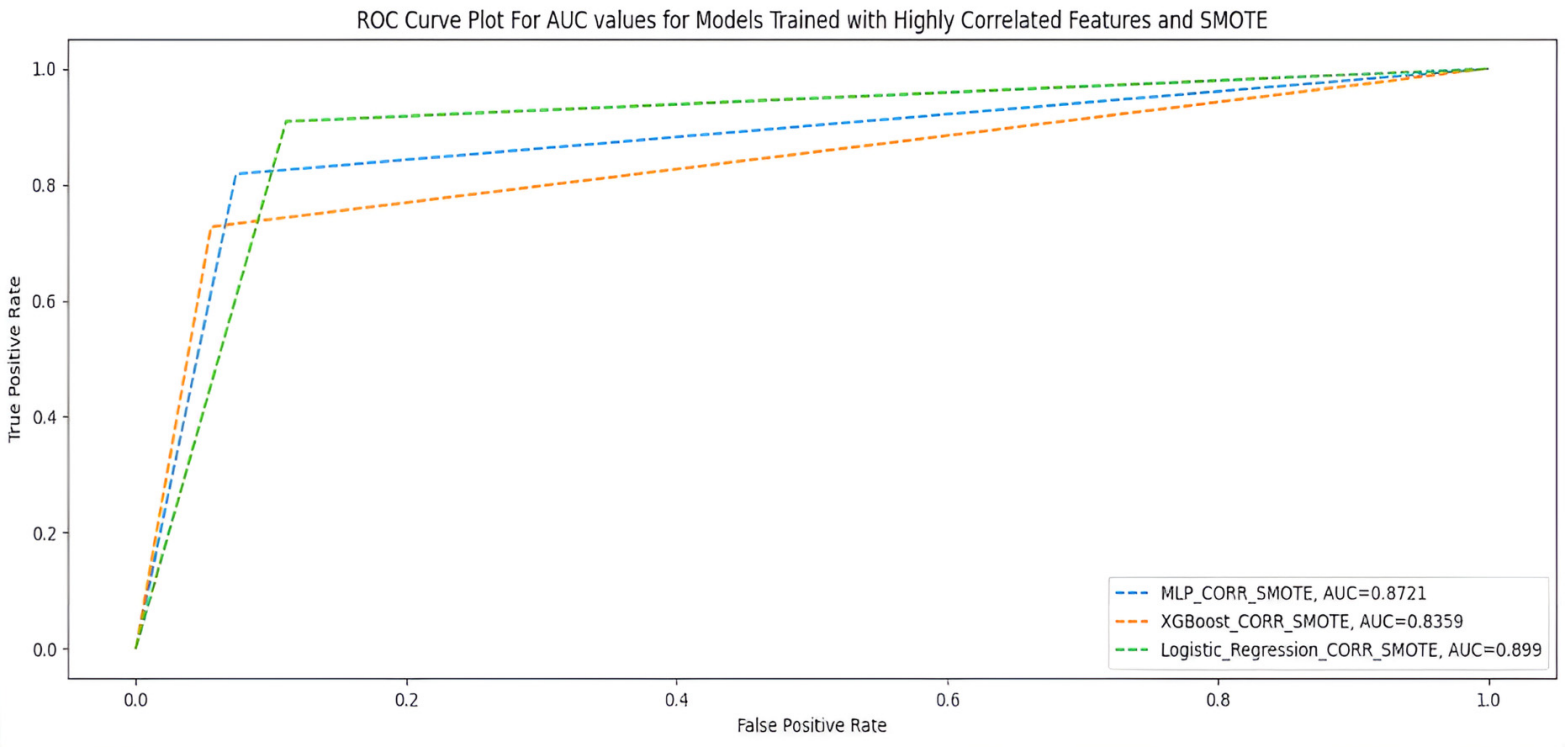
AUC scores for models trained with highly correlated features + SMOTE.

Table 7 shows the performance of the two ML models when trained with 23 highly correlated features with breakthrough infection with SMOTE. Figure 5 shows the AUC scores of the two models when trained under the same condition.

**Table 7.** Highly correlated features + SMOTE.

Measures	Deep MLP	XGBoost	Logistic regression
F1-score	0.91	0.91	0.90
G-Mean	0.87	0.83	0.90
MCC	0.70	0.67	0.69
AUC score	0.87	0.84	0.90
Accuracy	0.91	0.91	0.89

### All features using an imbalanced dataset

**Figure 6.**
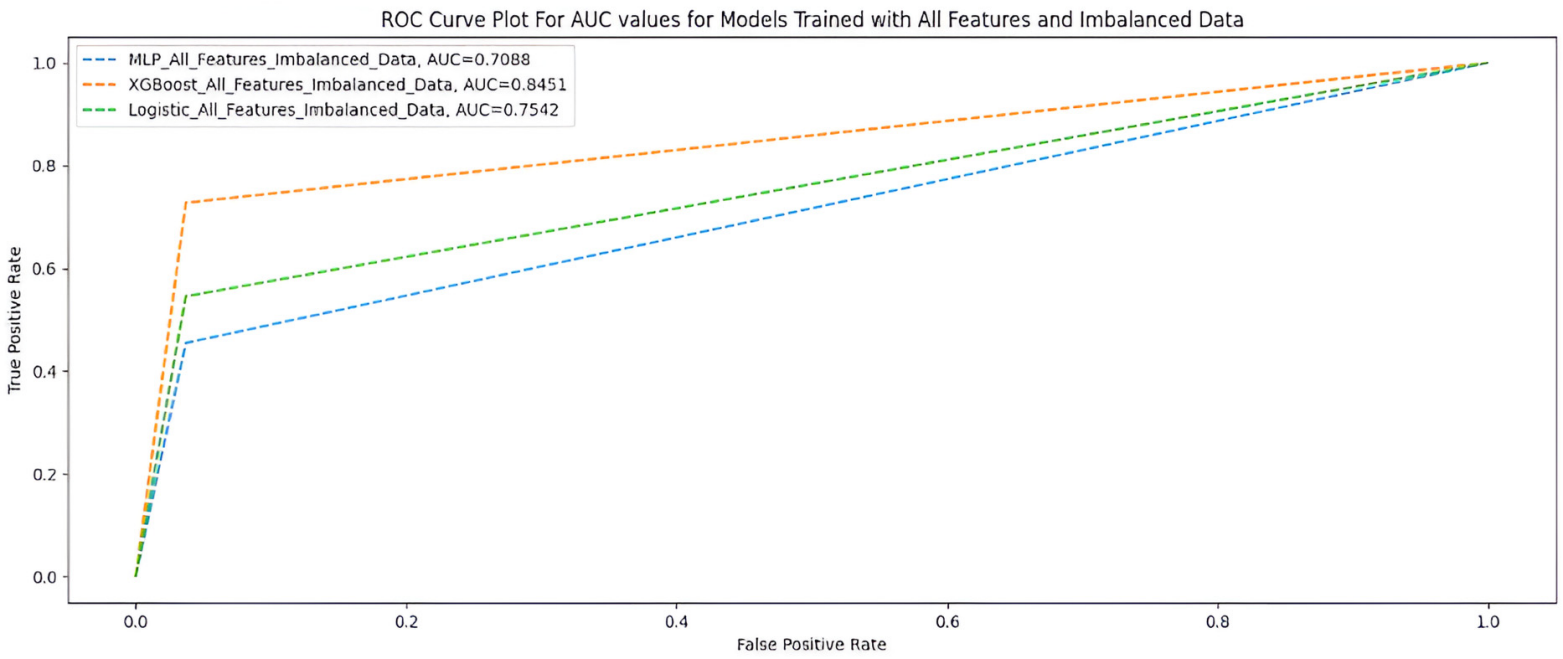
AUC scores for models trained with full features and imbalanced dataset.

Table 8 and Figure 6 show the performance of the three ML models when trained with all features using an imbalanced dataset.

**Table 8.** All features and imbalanced dataset.

Measures	Deep MLP	XGBoost	Logistic regression
F1-score	0.85	0.92	0.89
G-Mean	0.66	0.84	0.72
MCC	0.46	0.72	0.58
AUC score	0.70	0.85	0.75
Accuracy	0.86	0.92	0.89

### All features +  random oversampling

**Figure 7.**
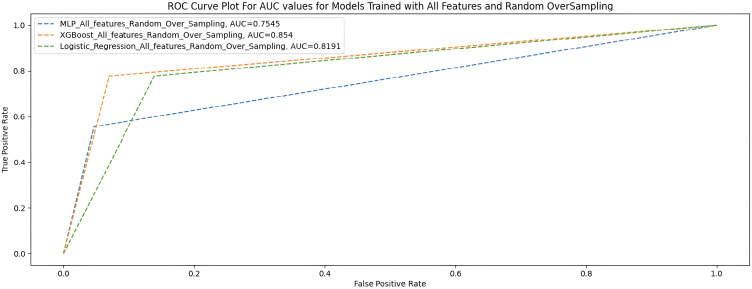
AUC scores for models trained with full features and random oversampling.

Table 9 and Figure 7 show the performance of the three ML models when trained with all features with random oversampling.

**Table 9.** All features + random oversampling.

Measures	Deep MLP	XGBoost	Logistic regression
F1-score	0.88	0.91	0.86
G-Mean	0.73	0.85	0.82
MCC	0.56	0.68	0.56
AUC score	0.75	0.85	0.82
Accuracy	0.88	0.90	0.85

### All features +  SMOTE

Table 10 shows the performance of the three ML models when trained with all features with SMOTE, and Figure 7 shows the AUC scores of the three models trained under the same treatments.

**Table 10.** All features + SMOTE.

Measures	Deep MLP	XGBoost	Logistic regression
F1-score	0.84	0.88	0.87
G-Mean	0.64	0.77	0.84
MCC	0.41	0.56	0.60
AUC score	0.69	0.78	0.84
Accuracy	0.85	0.88	0.86

The summary overview of the different experiments is shown in Table 11.

**Table 11.** Summary of models’ performance when trained with highly correlated features.

Highly correlated Features (imbalanced dataset)				Highly correlated features + random oversampling			Highly correlated features + SMOTE		
Metrics	DMLP	XGB	LR	DMLP	XGB	LR	DMLP	XGB	LR
F1-score	0.84	0.92	0.92	0.94	0.96	0.89	0.91	0.91	0.90
G-Mean	0.59	0.84	0.84	0.70	0.98	0.89	0.87	0.83	0.90
MCC	0.42	0.72	0.72	0.42	0.88	0.68	0.70	0.67	0.69
AUC score	0.66	0.85	0.85	0.92	0.98	0.89	0.87	0.84	0.90
Accuracy	0.86	0.92	0.92	0.94	0.96	0.88	0.91	0.91	0.89

The results in Tables 11 and 12 show that in terms of the F1 score and AUC score, the XGBoost had a generally better performance across all six treatments. The XGBoost model (F1 = 0.96 ± 0.02; G-Mean = 0.98 ± 0.01; MCC = 0.88 ± 0.001; AUC = 98 ± 0.001; accuracy = 0.96 ± 0.02) had the best performance of the three models when we used highly correlated features and all features in the dataset. The Deep MLP performed better when highly correlated features with ROT (F1 = 0.94 ± 0.04; G-Mean = 0.70 ± 0.01; MCC = 0.42 ± 0.01; AUC = 0.92 ± 0.001; accuracy = 0.94 ± 0.017) followed by the LR model, which performed well only when highly correlated features were used with non-SMOTE (F1 = 0.92 ± 0.08; G-Mean = 0.84 ± 0.01; MCC = 0.72 ± 0.01; AUC = 0.85 ± 0.001; accuracy = 0.92 ± 0.01).

**Table 12.** Summary of models’ performance when trained with all features.

All features (imbalanced dataset)				All features + Random oversampling			All features + SMOTE		
Metrics	DMLP	XGB	LR	DMLP	XGB	LR	DMLP	XGB	LR
F1-score	0.85	0.92	0.89	0.88	0.91	0.86	0.84	0.88	0.87
G-Mean	0.66	0.84	0.72	0.73	0.85	0.82	0.64	0.77	0.84
MCC	0.46	0.72	0.58	0.56	0.68	0.56	0.41	0.56	0.60
AUC score	0.70	0.85	0.75	0.75	0.85	0.82	0.69	0.78	0.84
Accuracy	0.86	0.92	0.89	0.88	0.90	0.85	0.85	0.88	0.86

### Complexity analysis of ML models

Usually, the computational complexity of algorithms is determined using the Big-Oh notation, which represents an upper bound of an algorithm's asymptotic complexity.^29^ Asymptotic algorithmic complexity is usually considered from two broad perspectives: time and space complexity. Time complexity measures the growth rate of time in relation to input data size. It computes the function O(n) (Big-oh notation), which is the measure of the order of time required by an algorithm to compute a task with input size *N*. Space complexity measures the order of resource (memory) storage needed by the algorithm to compute a task with input size *N*.

A ML algorithm's time and space complexity must be considered in terms of the learning complexity (the time to train a ML model) and inferential complexity (time to query a trained ML model).^30–32^ Thus, if there are *d* features in a dataset set of size *p*, then Tables 13 and 14 show the time complexity and space complexity of the three algorithms used in this study.

**Table 13.** Time complexity of the selected ML algorithms.

Algorithm	Training	Prediction
Deep MLP	O(PKmn^2^)	O(Kn^2^)
	A Deep MLP is a model of ANN with a dense architecture whose complexity can vary widely due to the number of hidden layers, the number of neurons in each layer, and the type of activation functions used in each layer. Thus, only a rough estimate is possible. The number of features (d) in a dataset and their types (numeric or categorical) influences the number of neurons/units (Ne) in the input layer of a Deep MLP (i.e., d ⇒ Ne) The Deep MLP has multiple inner(hidden) layers. Activations and weight updates will occur in all layers except the input layer. Thus, a MLP has a total of K layers. Typically, computation in an MLP involves the following: Matrix multiplication between neurons in an outer layer and an inner layer (u x v) will require quadratic time, which is O(n^2^) Activation function at each neuron in all layers of the network. If the number of neurons is m, the time complexity for activations in the MLP will be O(Km). The complexity of activations will depend on the choice of activation functions at specific layers. Simple activation functions like log or ReLU will occur in constant time, while more complex activations like Spline functions will require more time. Training an MLP entails using the backpropagation algorithm, which involves a forward pass and a backward pass (for weights update) over many epochs (P) (iterations) until the model is trained. During each forward pass computation involving all layers (K) in the Deep MLP, matrix multiplication and activations will take place; thus, the time complexity is O(Kn^2^ + Km) = O(Kmn^2^) During backward pass computation, activities similar to the forward pass will occur in all K layers. Thus, the time complexity is O(Kmn^2^) Thus, for P epochs, the time complexity is O(PKmn^2^)	MLP prediction only entails a forward pass computation; hence, the time complexity is O(Kmn^2^)
XGBoost	O(Tdnlog_2_n)	O(T log_2_n)
	XGBoost is an ensemble learning model consisting of decision trees (weak learners) and thrives on additive learning whereby the subsequent trees improve on the result of previous trees. The computation to construct a single binary tree requires O(nlog_2_n), while query time is O(log_2_n)^32^. For d features, the total computation in a decision tree will require O(dnlog_2_n). Considering that XGBoost has several decision trees, for T number of decision trees, the total computation will require O(Tdnlog_2_n)	The query time for one feature in a binary tree is O(log(n). For T trees, the time complexity will be O(T log_2_n)
Logistic Regression (LR)	O(dn)	O(d)
	LR Is a linear function that maps independent features (d_1,_d_2_.., d_j_) to a dependent feature/variable m, i.e., F: {d}→m. The time complexity for this operation is O(n) After that, a logistic (probability) value (p) is computed by using the Sigmoid function: p = 1/(1 + e^−m^) such that p = [0–1] The time complexity for computing the sigmoid function is O(1) because it will occur in constant time. Thus, for a dataset with d features, the time complexity of LR = O(dn)	This query instance involves one set of features of size d; hence, the computation will require O(d), where computation for each feature will occur in constant time O(1).

**Table 14.** Space (resource) usage complexity of ML algorithms.

Algorithm	Training	Prediction
Deep MLP	O(KN + W + B + D + F)	O(W + B + D + F)
	K = number of layers in the Deep MLP N = total number of neurons per layer W = weights matrices in architecture (set of weights between each adjacent layer) B = Bias values introduced in different layers of the architecture. F = Activation function outputs D = input data (D ⇒ d features) Thus, the space requirement during training can be estimated as O(KN + W + B + D + F)	The trained model needs to be stored, which requires the following: W = weights matrices in architecture (set of weights between contiguous layers) B = Bias values introduced in different layers of the architecture. F = Activation function values D = input data (D ⇒ d features) Thus, the space requirement can be estimated as O(W + B + D + F)
XGBoost	O(TV + TU)	O(TV + TU)
	T = number of decision trees in the ensemble model V = total number of nodes per decision tree U = values stored in the leaf nodes of a decision tree Thus, the space requirement during training is O(TV + TU)	T = number of decision trees in the ensemble model V = total number of nodes per decision tree U = values stored in the leaf nodes of a decision tree Space requirement during testing is O(TV + TU)
LR	O(nd)	O(d)
	All d features have to be stored in memory to train the model. Hence, O(nd) is required.	Only the query instance must be stored in memory to test the model. Hence, O(d) is required.

### Analysis of feature importance generated by ML models based on SHAP values

**Figure 8.**
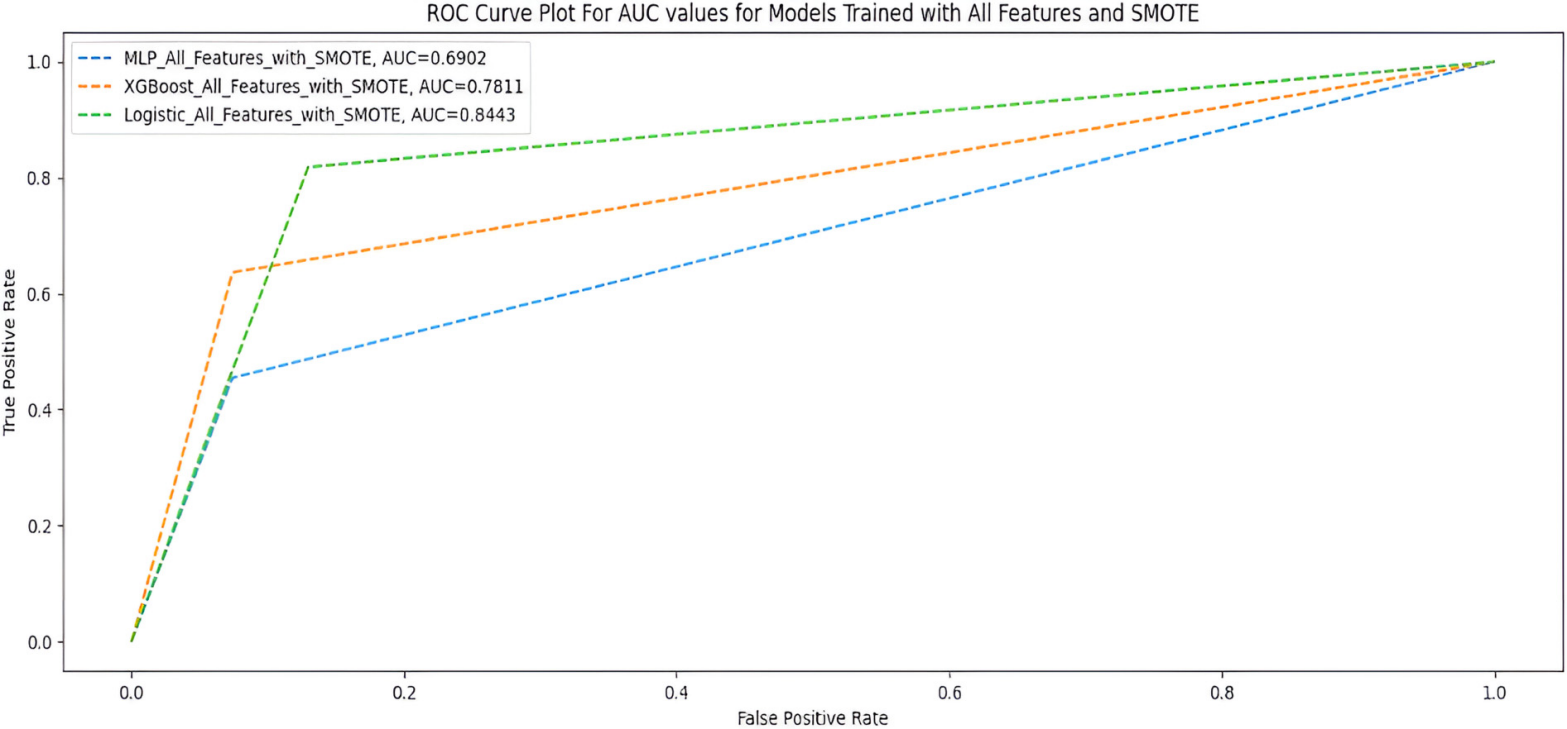
AUC scores for models trained with full features + SMOTE.

**Figure 9.**
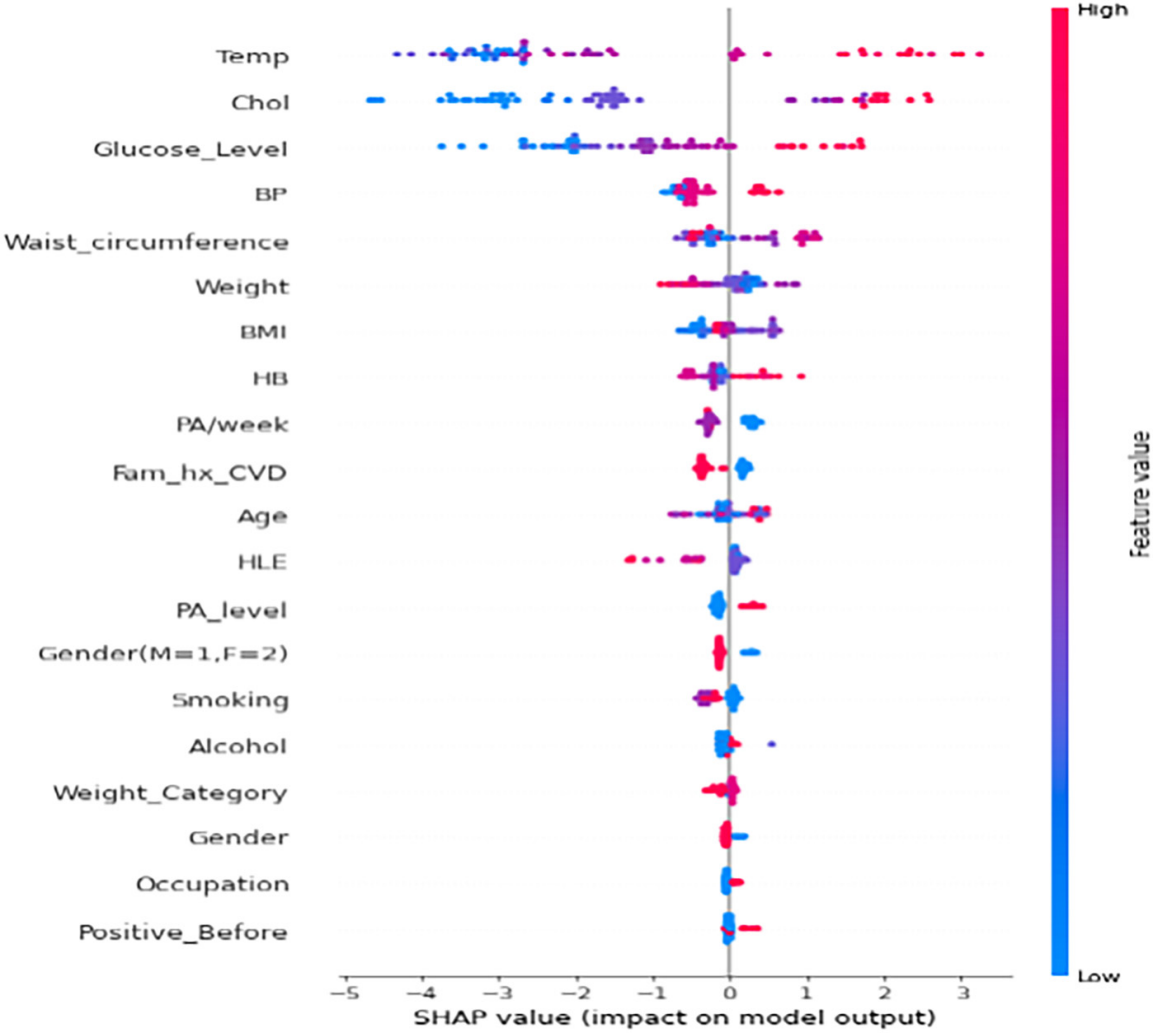
Important features of XGBoost based on SHAP values using summary plot.

Based on the results of the experiments, we selected the instance with the best performance. We identified the features that had the most influence on their prediction (feature importance) based on SHAP values. We selected the XGBoost model when the highly correlated features plus ROT were used. The relative importance of features based on SHAP values is shown in Figures 8 and 9.

**Figure 10.**
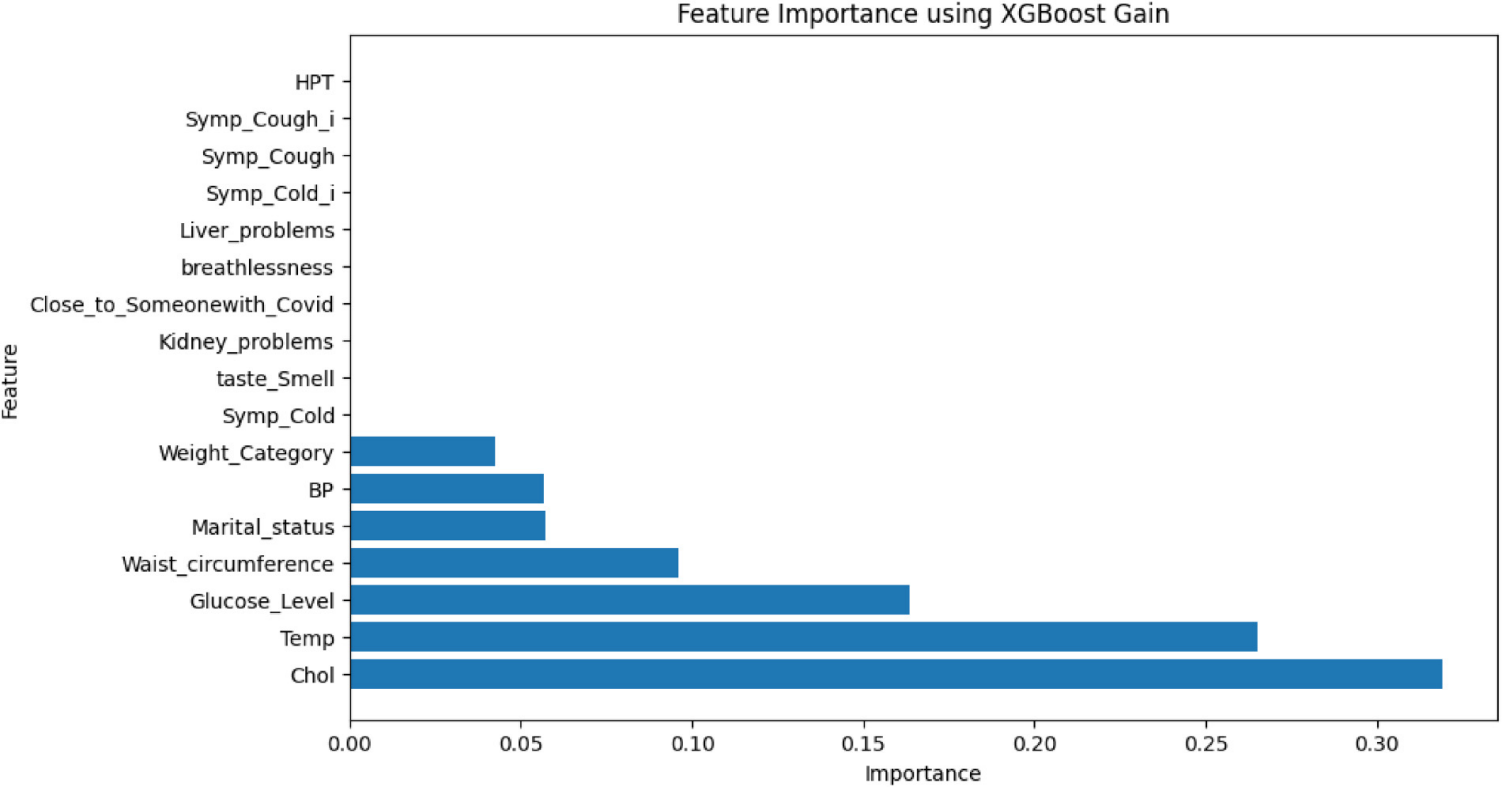
Feature importance using XGBoost gain.

From the results in Figure 9, the nine most critical variables for predicting COVID-19 breakthrough infection in patients were *body temperature, total cholesterol, glucose level, blood pressure, waist circumference, weight, BMI, haemoglobin, and physical activity per week (PA/Week).* We also used the XGBoost Gain method to determine feature importance (see Figure 10) and found that the most important features generated were the same as those obtained from SHAP. From the feature importance generated from SHAP (Figure 8), we could infer that generally, lower values (indicated in blue colour) of *body temperature, total cholesterol*, *glucose level, blood pressure level, and BMI* lead to reduced chances of breakthrough infection. Also, lower values (indicated in blue colour) of *PA/Week* lead to increased risk of breakthrough infection, while higher values of *PA/Week* will lead to reduced risk of breakthrough infection. In addition, higher values (indicated in red colour) of *weight*, *waist circumference, and haemoglobin level* will lead to a higher risk of breakthrough infection.

## Discussion

Based on the results of our study, we learned the following about the three selected ML models:

### The performance of Deep MLP

Nested cross-validation can enhance the performance of Deep MLP if the proper training parameters are selected. The Deep MLP had a generally acceptable performance in terms of F1-score and AUC when highly correlated features were used for training with random oversampling (F1 = 0.94 (very good); AUC = 0.92 (very good)). This observation means that the Deep MLP performed best when highly correlated features were used with ROT followed by when SMOTE was applied (see Table 11). The G-Mean score (0.70) and MCC (0.42) were also fair in terms of rating. The G-Mean corresponds again with a better prediction of the breakthrough infection shown in Table 7 during this treatment. According to Kubat,^33^ the G-Mean considers the relative balance of the classifier's performance on both the infected and the non-infected classes. It is defined as a function of the classifier's sensitivity and specificity. Again, MCC is a measure from the field of Bioinformatics, where class imbalance occurs very often. It is an adaptation of the Pearson correlation coefficient to evaluate the correlation in confusion matrices. MCC ranges from −1 (when the classification is always wrong) to 0 (when it is no better than random) to 1 (when it is always correct).^27^ Therefore, a G-Mean score of 0.70 (good) and MCC of 0.42 (fair) for the Deep MLP was a relatively good model performance. We also observed that the Deep MLP had a significantly better G-Mean score (0.87) and MCC (0.7) when SMOTE was used. The Deep MLP has greater computational complexity (see Tables 13 and 14) in terms of training time and space (resource usage) when compared to the LR. Its strength, however, lies in its propensity to grow in performance with large and complex datasets, although it would mean needing more hardware resources and training time.

### Performance of XGBoost

XGBoost is one of the most powerful ML algorithms, particularly when dealing with tabular (non-image) data. It is acclaimed for being successful in several Kaggle competitions. Based on our experimentation, we found that XGBoost (F1 = 0.96 (very good); AUC = 0.98 (very good)) performed best when used with the highly correlated features with ROT. In this case, XGBoost outperformed the Deep MLP and LR. Furthermore, XGBoost recorded a higher G-Mean (0.98) and MCC (0.88) than the Deep MLP and LR in all cases. This shows that the XGBoost outperformed the other two models in dealing with imbalanced data.

Moreover, the improved prediction of the breakthrough infection in Tables 11 and 12 confirms this notion. Like the Deep MLP, XGBoost is an ensemble model with a big architecture consisting of a huge number of decision trees, which can also increase its computational complexity. But it can be very efficient in dealing with large datasets. Thus, for a very large dataset, it would be a strong candidate for consideration.

### Performance of LR

LR has proved to be a potent classifier, particularly for linearly separable datasets with simple relationships among the variables.^34^ Thus, in many cases, LR is used as a baseline to assess the performance of more complex ML algorithms. When we used highly correlated features with ROT, we found that LR (F1 = 0.89 (good); AUC = 0.89 (good); G-Mean = 0.89 (good); MCC = 0.68 (good)) had generally good performance. Although it had lower scores compared to the Deep MLP in terms of AUC and F1-scores, it would be more reliable in terms of sensitivity and specificity because of its higher G-Mean and MCC scores compared to the Deep MLP (G-mean = 0.7; MCC = 0.42). Thus, the LR will be a good choice for a small or medium-sized dataset for predictive modelling. However, complex ML algorithms like Deep MLP (ANN) and XGBoost will perform much better with more complex datasets having non-linear relationships. The LR is a very simple algorithm that does not have high computational complexity in terms of time and space (Tables 13 and 14). This makes LR suitable for use when dealing with simple datasets and the availability of minimal hardware resources.

### The effect of ROT and SMOTE on ML models

The use of ROT and SMOTE did not significantly improve the performance of the Deep MLP regarding the six options we tested. The Deep MLP showed a slight improvement when the highly correlated features and all features in the dataset were used to train the model. This might be because the dataset needed to be bigger, making the difference insignificant. By comparing Deep MLP with the XGBoost and the LR, we found that applying SMOTE did not significantly change model performance. However, with ROT, model performance was significantly improved, especially when we trained MLP and XGBoost models with highly correlated features. The improvement from applying the two data resampling techniques (SMOTE and ROT) was more evident in the higher G-mean and MCC scores of ML models than when an imbalanced dataset was used. This suggests that the classifiers have an enhanced ability to distinguish between the distinct classes and produce more reliable and consistent predictions.

Generally, the performance of the three ML models compares favourably with the results of the few cohort studies where ML has been used to predict COVID-19 breakthrough infection or reinfection reported in the literature.

### Lessons from COVID-19 breakthrough-infection prediction

The most critical variables for the prediction of breakthrough infection (see Figure 9) are (1) *body temperature,* (2) *blood total cholesterol level*, (3) *blood glucose level*, (4) *blood pressure level*, (5) *waist circumference,* (6) *body weight*, (7) *BMI*, (8) *haemoglobin level*, and (9) *PA/Week.* The results concur with the positions reported by other researchers.^35–39^ According to Radenkovic et al.,^35^ breakthrough infections follow a more severe clinical course in patients with CVD, hypertension, and overweight/obesity. Stefan^36^ observed that obesity is a significant risk factor that promotes vaccine-breakthrough SARS-CoV-2 infections in fully vaccinated people. Aparisi et al.^37^ found that low-density lipoprotein cholesterol (LDL-c) serum levels are independently associated with higher 30-day mortality in COVID-19 patients. Specifically, the authors found that LDL-c ≤ 69 mg/dl, C-reactive protein > 88 mg/dl, and lymphopenia <1000 at admission were independently associated with 30-day mortality. Also, Kočar et al.^38^ reported that cholesterol is recognised as a molecule regulating the entry of the SARS-CoV-2 virus into the host cell. Several researchers have confirmed that diabetes is one of the most important comorbidities linked to the severity of all three known human pathogenic coronavirus infections, including severe acute respiratory syndrome coronavirus. Some authors^39–41^ observed that obese individuals were at higher risk of developing complications from SARS-CoV-2. Woods et al.^25^ opined that the immobilisation and the physical inactivity of patients could down-regulate the ability of organ systems to resist viral infection and increase the risk of damage to the immune, respiratory, cardiovascular, and musculoskeletal systems and the brain. Shahidi et al.^42^ concurred with Woods et al.^25^ by reporting that physical activity benefits include musculoskeletal and cardiovascular health, healthy body weight, and neuromuscular awareness, which would help the body fight the virus. These previous findings from the literature confirm that the nine variables identified by our prediction models have prognostic value regarding breakthrough infection in vaccinated individuals. These variables (*body temperature, total cholesterol, glucose level, blood pressure, waist circumference, weight, BMI, haemoglobin, and PA/Week*) are good indicators of the likely existence of comorbidities such as obesity, diabetes, hypertension, other cardiovascular diseases, and poor lifestyle habits that increase the probability of COVID-19 breakthrough infection for persons living with one or more of these comorbidities.

### Limitations of the study

A limitation of this study is the relatively small size of the dataset. The data used for the study were from direct interaction (examination and interview) with the participants who enrolled within the study period, which means fewer samples were available. It would have been different if we extracted our data from hospital records, which was not our objective in this study. For this reason, the findings of this study have limited generalisability in that the result may vary when applied to different datasets or healthcare settings. For example, it may be possible for the three selected ML models to exhibit other characteristics in terms of their performance metrics. Thus far, very few case studies about the potency of COVID-19 vaccination using ML from the African context have been reported in the literature, which makes our experimentation and findings valuable. Our results also align with several clinical studies on identifying the critical variables of COVID-19 breakthrough infection (see Table 15).

**Table 15.** Overview of studies on prediction of COVID-19 breakthrough infection or reinfection using ML models.

Author	Dataset	Accuracy (%)	F1 (%)	AUC (%)	G-Mean (%)	MCC (%)
This study						
Deep MLP	The study used 23 highly correlated predictors with Nested cross-validation, SMOTE, and ROT as treatments. The dataset consists of 257 samples.	94.0	94.0	92.0	70.0	42.0
XGB		96.0	96.0	98.0	98.0	88.0
LR		88.0	89.0	89.0	89.0	68.0%
Ahamad et al.^19^					Precision	Recall
RF	The study predicted COVID-19 reinfection using a dataset of patients who complained of adverse reactions after vaccination as extracted from the Vaccine Adverse Event Reporting System (VAERS) of observed individuals from December 2020 to 16 February, 2022. Data from a total of 11,266 individuals with severe reactions was used.	100.0	100.0	100.0	96.0	96.0
LGBM		99.0	99.0	98.0	94.0	91.0
SVM		99.0	99.0	98.0	82.0	67.0
DT		99.0	99.0	99.0	96.0	94.0
XGB		99.0	99.0	99.0	95.0	93.0
GBM		93.0	95.0	90.0	81.0	79.0
Ebrahimi et al.^20^					Overall Survival (OS)	
Elastic-net Regularized Cox-adjusted PH + Backward Stepwise Elimination	Two ML algorithms were applied: elastic-net regularised Cox-adjusted PH model and backward stepwise elimination to a dataset of 283 reinfected COVID-19 patients admitted to 26 medical centres in Iran.	–	–		Using the Kaplan-Meier approach, the overall survival (OS) at 95% confidence interval (CI) that was obtained for days 7, 14, and 21 were 87.5%, 78.3%), and 52.2% respectively.	
Afrash et al.^21^					Sensitivity	Kappa Statistic
HGB	The study used a dataset of 870 re-admitted COVID-19 patients. The LASSO algorithm was used to select 14 features out of 42 features as the most relevant predictors. The study also found that COVID-19 status, ICU admission, and oxygen therapy were the features most associated with readmission of COVID-19 patients.	81.76	82.01	82.33	82.96	82.40
BC		84.70	84.30	84.30	84.70	84.36
MLP		88.60	88.10	88.20	88.40	88.60
SVM: Kernel		88.96	89.20	89.20	91.20	88.70
SVM:RBF		85.70	85.90	86.30	86.10	86.70
XGB		91.70	91.80	91.45	91.60	91.37
KNN		88.35	89.20	88.60	89.20	88.3
Chen et al.^22^						
Visualised nomogram using the stepwise multivariate logistic regression algorithm	The study used a dataset of 6189 vaccinated individuals, consisting of COVID-19 test-positive cases (*n* = 219) and test-negative controls (*n* = 5970) during the outbreak of the Delta variant in September 2021 in Xiamen and Putian cities, Fujian province of China.	-	-	83.8%	-	-

MLP: multilayer perceptron; RF: random forest; SVM: support vector machines; XGB: extreme gradient boosting; DT: a decision tree algorithm; LR: logistic regression; NB: Naïve bayes; LGBM: light gradient boosting machine; ANN: artificial neural networks; GB: gradient boosting; GBM: gradient boosting machine; HGB: Hist gradient boosting; BC: bagging classifier.

## Conclusion

The results presented in this study can help guide medical practitioners and patients in reducing the chances of breakthrough infections. Since none of the people was ill at the time of sample collection, the positive correlations between the detection of SARS-CoV-2 and expected symptoms, such as a higher body temperature, total cholesterol and glucose level, haemoglobin, and BMI, are considered clinically relevant. This relates to the results obtained from the multiple diagnostic and predictive testing methods that underpin our unique data set, which were harnessed through AI to facilitate COVID-19 detection, treatment, and management.

In future work, we shall explore the feasibility of implementing a novel multimodal diagnostic and predictive model for COVID-19 and its associated comorbidities for sub-Saharan Africa (SSA), as described in our concept paper.^8^ The results obtained in this study provide the clinical context for incorporating COVID-19 host-genetics into a pathology-supported genetic testing (PSGT) framework for translating population risk into personal utility.^43^ Daramola et al.^8^ state that AI technology can augment sound decision-making using PSGT in patients stratified by BMI and other forms of data (text, image, audio). Thus, the combination of AI and PSGT can be a viable tool to supplement the capabilities of healthcare systems in SSA in the fight against COVID-19, other highly prevalent infectious diseases, and future pandemics. In addition, we plan to create a decision support tool (web app) that can guide medical practitioners when dealing with critical cases of COVID-19 and other prevalent infectious diseases.

## Appendix

**Appendix 1.** Results obtained from imputation using features with 50% or less missing values and *nested cross-validation.*

Highly correlated features										All features								
Metrics	Non-SMOTE			Random oversampling			SMOTE			Non-SMOTE			Random oversampling			SMOTE		
	MLP	XGB	LR	MLP	XGB	LR	MLP	XGB	LR	MLP	XGB	LR	MLP	XGB	LR	MLP	XGB	LR
Acc	0.83	0.85	0.85	0.88	0.88	0.83	0.82	0.86	0.83	0.85	0.86	0.86	0.88	0.85	0.85	0.86	0.86	0.82
Recall	0.83	0.85	0.85	0.88	0.88	0.83	0.82	0.86	0.83	0.85	0.86	0.86	0.88	0.85	0.85	0.86	0.86	0.82
F1	0.82	0.85	0.84	0.89	0.88	0.85	0.83	0.86	0.85	0.83	0.86	0.85	0.88	0.84	0.86	0.85	0.86	0.82
AUC	0.64	0.73	0.69	0.83	0.78	0.90	0.74	0.74	0.83	0.65	0.74	0.70	0.78	0.65	0.86	0.70	0.74	0.71
G-score	0.58	0.70	0.65	0.76	0.76	0.89	0.74	0.71	0.83	0.59	0.71	0.66	0.76	0.59	0.86	0.66	0.71	0.69
MCC	0.33	0.45	0.41	0.50	0.56	0.61	0.43	0.49	0.55	0.37	0.49	0.46	0.56	0.35	0.58	0.46	0.49	0.39

**Table:** Results obtained from imputation using features with 30% or less missing values and *nested cross-validation.*

Highly correlated features										All features								
Metrics	Non-SMOTE			Random oversampling			SMOTE			Non-SMOTE			Random oversampling			SMOTE		
	MLP	XGB	LR	MLP	XGB	LR	MLP	XGB	LR	MLP	XGB	LR	MLP	XGB	LR	MLP	XGB	LR
Acc	0.89	0.92	0.92	0.92	0.96	0.88	0.89	0.89	0.88	0.88	0.92	0.89	0.88	0.90	0.85	0.88	0.89	0.80
Recall	0.89	0.92	0.92	0.92	0.96	0.88	0.89	0.89	0.88	0.88	0.92	0.89	0.88	0.90	0.85	0.88	0.89	0.80
F1	0.89	0.92	0.92	0.93	0.96	0.89	0.90	0.89	0.89	0.87	0.92	0.89	0.88	0.91	0.86	0.87	0.89	0.81
AUC	0.75	0.85	0.85	0.91	0.98	0.89	0.86	0.79	0.93	0.71	0.85	0.75	0.80	0.85	0.82	0.74	0.79	0.70
G-score	0.72	0.84	0.84	0.87	0.98	0.89	0.86	0.78	0.93	0.66	0.84	0.72	0.79	0.85	0.82	0.72	0.78	0.68
MCC	0.58	0.72	0.72	0.70	0.88	0.68	0.66	0.60	0.70	0.51	0.72	0.58	0.60	0.68	0.56	0.53	0.60	0.36
